# Clinical remission off medication in greek adults with juvenile idiopathic arthritis during a 17 year follow-up period

**DOI:** 10.1186/1546-0096-12-S1-P152

**Published:** 2014-09-17

**Authors:** Despoina Dimopoulou, Maria Trachana, Polyxeni Pratsidou-Gertsi, Prodromos Sidiropoulos, Athina Theodoridou, Foteini Lada, Alexandros Garyphallos

**Affiliations:** 1 4th Dept of Internal Medicine, Aristotle University of Thessaloniki, Hippokration Hospital, Thessaloniki, Greece; 2 1st Dept of Pediatrics, Aristotle University of Thessaloniki, Pediatric Immunology and Rheumatology Referral Center, Hippokration Hospital, Thessaloniki, Greece; 3 Dept of Rheumatology, University of Heraklion, Heraklion, Greece

## Introduction

Clinical remission off medication (CR) in patients with Juvenile Idiopathic Arthritis (JIA) is the optimal aim of treat -to -target strategies. No relevant data have been published for Greek young adults so far.

## Objectives

To assess the achievement of CR and identify CR’s predictors in adults with JIA over a-long-term disease course.

## Methods

JIA patients ≥ 18 years, and a ≥5 years disease duration were enrolled in this longitudinal retrospective cohort study. Radiographic damage was based on total modified Sharp/van der Heijde score (TmSvdHS), articular and extra-articular damage on JADI and physical ability on HAQ-DI.

## Results

98 patients (69 females) with a mean age at disease onset of 7.8 years, an interval from onset to last visit of 17.1 years and a current age of 24.9 years were studied. 37.8% achieved ≥ 1 episode of CR and 21.6% ≥2. The 7 JIA subtypes differed in respect to CR attainment (p=0.008), the worst being patients with polyarthritis RF positive (0%) and the best those with persistent oligoarthritis (87.5%). In 51.4% of them CR lasted for ≥5 years. Gender, age at disease onset, ANA and anti-CCP positivity were not correlated with CR. CR duration was significantly correlated with lower JADI-A (p=0.008), JADI-E (p<0.001), TmSvdHS (p=0.002) and HAQ-DI (p=0.018), while predictors of shorter CR state were polyarticular subtype (p=0.004) and longer duration of disease activity within the first 5 years (p=0.001).

## Conclusion

Shrinking of disease activity periods in long-term JIA induced by improved treatments leads to extended CR periods and avoids structural damage and physical disability.

## Disclosure of interest

None declared.

